# Applying and validating a quality management system for in-house developed medical software

**DOI:** 10.3389/fdgth.2025.1461107

**Published:** 2025-04-01

**Authors:** Vera Lagerburg, Michelle van den Boorn, Reinier F. Crane, Koen Welvaars, Jaap M. Groen

**Affiliations:** ^1^ Department of Medical Physics, OLVG, Amsterdam, Netherlands; ^2^ Department of Medical Physics and Instrumentation, St. Antonius Hospital, Nieuwegein, Netherlands; ^3^ Department of Intensive Care Unit, OLVG, Amsterdam, Netherlands; ^4^ Analytics Department - Data Science Team, OLVG, Amsterdam, Netherlands

**Keywords:** medical software, quality management system (QMS), medical device regulation (MDR), in-house development, ISO13485

## Abstract

**Introduction:**

The legislation regarding in-house development of medical devices has changed substantially with the introduction of the Medical Device Regulation (MDR) in 2021. Practical guidelines regarding the implementation of a quality management system for in-house developed medical software are scarce. In this article, we describe our experience with fulfilling the requirements of the MDR for an in-house developed prediction model, qualified as medical software.

**Methods and materials:**

Our quality management system (QMS) is based on the ISO13485:2016. It is a workflow consisting of elements subdivided in subelements, which consist of procedures, work instructions and/or formats. Within the data science team procedures regarding the process and documentation of software development were already in place. The existing procedures and documentation were compared with the procedures of the QMS and where possible, integrated into the workflow. The gap between the existing procedures regarding software development and the procedures of the QMS was defined. Existing documentation and procedures were used as much as possible. If there was a gap, additional documentation was written.

**Results:**

The majority of the (sub)elements was considered to be applicable for our software development project beforehand. Only in 6 out of 32 cases (19%), the (sub)element was deemed not applicable. For 32% of the applicable elements the documentation of the data scientists team was sufficient and additional information was not needed. For 23% the documentation was incomplete and we decided to add relevant information to fulfil the requirements of the MDR and for 45% the documentation was completely lacking and the standard formats were used.

**Conclusion:**

We showed in this article that it is possible to use a QMS developed with physical medical products in mind for medical software and thus comply with applicable legislation and regulations. This can be done without too much effort when there is already some structured form of software development methodology in place.

## Introduction

The legislation regarding in-house development of medical devices has changed substantially with the introduction of the Medical Device Regulation (MDR) in 2021. In its predecessor, the Medical Device Directive (MDD), in-house development of medical devices was not regulated at all. In the MDR a special article regarding in-house development was added (1). This article, article 5.5, describes the requirements an in-house developed medical device must fulfill, of which an important one is that the manufacturing and the use of the devices occurs under an appropriate quality management system (QMS). What constitutes an appropriate quality management system is not specified. The Medical Device Coordination Group has written some guidance documents to further explain the MDR. Regarding the definition of an appropriate quality management system, they state that ‘article 10(9) of the MDR can be used as a guidance (with some MDR-specific exceptions) on what an appropriate QMS for manufacturing in-house devices is. If applicable, ISO-standards concerning for instance manufacturing and risk management can be used, especially if harmonized with the MDR’ (2).

ISO13485 (3) is a standard for a QMS that is often used in companies that develop medical devices. Both the MDD and the ISO13485 were originally written with physical medical devices in mind. In the first version of the MDD from 1993 software was only mentioned twice, viz. in the definition, and in the last update of 2007 only two statements regarding medical software were added (4). In the MDR medical software is a more substantial part, but the regulation is still more oriented towards physical medical devices, shown by a number of essential requirements that are not applicable for software, e.g., requirements regarding cleaning, packaging and labelling (1).

Practical guidelines regarding the implementation of the MDR for in-house developed medical devices and especially medical software are scarce. The literature regarding QMSs that are in place mostly describe QMSs developed for physical medical devices. Willemsen et al. (5, 6) describe a case study of the regulatory approval of an in-house developed surgical implant. QMSs described in literature for in-house development of medical software are mostly only theoretical. Bartels et al. (7) describe their perspective on a quality system for an AI-based decision support model, where they applied a QMS that mimics the ISO15189 used in medical laboratories. Especially for Artifical Intelligence Medical Software (AIMS) challenges appear (8), because one of the key properties of AIMS is that it improves itself, which makes the required pre-market validation a challenge. Zanca et al. (9) and Beckers et al. (10) provide practical guidelines on regulatory aspects for AIMS. They describe some relevant standards, e.g., the IEC62304 and the EN82304-1, and all the steps that should be taken to safely implement AIMS including the challenges. Niemiec et al. (11) give some guidelines for classification of medical AI devices and refer to upcoming guidance documents and regulation, such as the European AI act and guidance documents for further clearance of the regulation.

Practical guidelines and experience with the implementation of a quality management system for in-house developed medical software are scarce. At our hospital, we develop medical devices, most of which are medical instruments or 3D printed surgical guides. We have set up a quality system, based on the requirements of the MDR and our experience with this type of products, but with the intention to be appropriate for all types of medical devices, including medical software. Recently we started with the development of prediction models, which qualify as medical devices according to the MDR. Because most QMSs are based on physical products, we decided to test and validate our QMS on an in-house developed prediction model.

In this article, we describe our experience with fulfilling the requirements of the MDR for an in-house developed prediction model, qualified as medical software.

## Methods and materials

### Quality management system

In our hospital, a QMS is in place. This QMS was implemented to be used for all in-house developed medical devices, including medical software. To align with the requirements of the MDR, we based our quality management system on the ISO13485:2016 (3). Our QMS is a workflow consisting of elements subdivided in subelements which consist of procedures, work instructions and/or formats whichever is appropriate for that (sub)element (Table 1). Two examples of formats can be found in the supplementary material (Supplementary 1, 2). Risk analysis formats were developed in accordance with the ISO14971:2007-03 (corrected and reprinted 2012-07) (12). Until now, this QMS was only used for physical medical products.

**Table 1:** Elements and subelements of our QMS for the in-house development of (physical) medical devices.

Number	Element	Subelements	Short description	Procedure	Work instruction	Format
1	Request		Request from clinic to develop a medical device, including the requirements	x		
1.1		Unique device identifier			x	
1.2		Request form				x
1.3		Requirements			x	x
2	Consideration		Consideration if the medical device will be developed in-house	x	x	
2.1		Market exploration			x	
3	Define design criteria		Definition of the design criteria	x		x
4	Risk analysis		Risk management plan consisting of a risk analysis following ISO14971 and a risk analysis specific for the risks during use	x		
4.1		Risk analysis ISO14971				x
4.2		Risk analysis use		x		x
4.3		Risk management plan			x	x
5	Design		Definition of the design process	x		
5.1		Schematic design				
5.2		Design process				
6	Design verification		Verification of the design (does the design match the design criteria)	x		x
7	Prototype		Development of a prototype	x		
7.1		Photo				
8	Define testprotocol		Definition of a testprotocol	x		x
9	Define preconditions		Definition of all preconditions that should be fulfilled before using the medical device	x		
9.1		List of all preconditions				x
9.2		Required training for administrators				x
9.3		Required training for users				x
9.4		Cleaning and sterilization requirements				
9.5		Maintenance prescription				x
9.6		Installation prescription				x
9.7		User manual				x
9.8		Packaging				x
9.9		Label				x
9.10		Transporting prescription				x
10	Validation		Validation if the prototype matches the request	x		x
11	Clinical research		Research	x		
11.1		Research plan				x
11.2		Research results				
13	Production		Production	x		
14	Post market surveillance		Follow-up after commissioning of the medical device	x		
14.1		Post market plan			x	x
14.2		Post market reports				x
15	Technical file		Technical file, including all essential requirements			
15.1		Technical file			x	x
15.2		Essential requirements				x
15.3		Additional requirements				
16	Declaration		Declaration of conformity			x
17	Handover protocol		Declaration of the handover of the medical device to the user			x

### Software development

In our hospital (medical) software is developed by the data science team. This team uses the CRISP-DM method (13) to develop software.The CRISP-DM methodology was developed prior to the introduction of the MDR and gives a pragmatic approach for software development The CRISP-DM methodology is an iterative process with six process steps: business understanding, data understanding, data preparation, modelling, evaluation and deployment (14), resulting in multiple documents describing different aspects of the software and the software development process. This documentation is subsequently summarized on an internal webportal (Microsoft Devops Wikipedia), to be able to retrospectively look at choices made with associated conclusions and assumptions.

### Practical application of the QMS

During the development of a prediction model, classified as a medical device, we decided to apply the available QMS, in combination with the documentation provided using the CRISP-DM method, and test if in this way we could fulfill the requirements of the MDR. A team consisting of a data scientist, two medical doctors, a technical physician and two medical physicists developed the prediction model. The technical physician and one of the medical physicists were responsible for following the requirements of the QMS and the companion documentation. Every member of the development team gave input for the documentation, depending on his or her expertise. Both medical doctors were responsible for documentation regarding all clinical input, e.g., which data is clinically relevant and which not, what sensitivity and specificity of the model was required and how the clinical implementation was performed. The data scientist and one of the medical physicist together documented the design and validation the prediction model.

After deciding on each other's responsibility regarding the documentation, the next step was to determine which elements of the QMS workflow were applicable for the development of medical software in general and which steps were applicable for our prediction model specifically. This was done by discussing each element within our team. We also decided who was responsible for delivering the concurrent documentation, based on the expertise of each team member and their role in the project.

Within the team of the data scientist procedures regarding the process and documentation of software development were already in place. These procedures were used as the basis for the documentation in this process. The existing procedures and documentation were compared with the procedures of the QMS and where possible, integrated into the workflow. The gap between the existing procedures and documents regarding software development and the procedures and documents of the QMS was defined. This was done in two steps. First, the required documentation within the CRISP-DM method was compared to the required documentation of the QMS. When the QMS required documentation was not available within the CRISP-DM methodology, the documentation was stated missing. If the documentation was available, the content of the CRISP-DM documentation was compared to the content requirements of the QMS. One of the medical physicists and the technical physician performed these analyses. Existing documentation and procedures were used as much as possible. If there was a gap additional documentation was written. We scored the percentage of documents that were available and complete within the data scientists’ team documentation, partly available or totally lacking.

During each step in the development of the prediction model, the required documentation was recorded. The prediction model was developed in the Azure Machine Learning studio within the workspace of OLVG. After evaluating to a safe and strong performing model, it was implemented in the hospital front-end environment (Epic Hyperspace, Epic Systems Corporation). In development and implementation the standard Epic developer's tool and environments were applied.

### External audit

In a later stage, an external MDR expert audited our complete QMS. The main purpose of this external audit was to verify the implementation of the QMS, by assessing the current processes and check if the QMS fulfilled the requirements of the ISO13485:2016. During theaudit some of our technical files were reviewed, including the file of the prediction model. This gave additional insight about the suitability of the QMS for medical software.

## Results

The majority of the (sub)elements was considered to be applicable for our software development project beforehand. Only in 6 out of 32 cases (19%), the (sub)element was deemed not applicable (Table 2, column 3). However, during collection of the required documentation, another four subelements were also considered not to be applicable for this specific software development project (Table 2, column 4). An explanation for the inapplicability of these items is given in Table 2, column 5.

**Table 2:** Overview of the subelements of the quality management system that were deemed applicable for the in-house developed software, the responsible person for the documentation and if the already available documentation and/or formats could be used.

Element	Subelements	Applicable for medical software yes/no	Applicable for our prediction model yes/no	Explanation	Responsible for documentation	Documentation of the data scientist team fulfilled requirement yes/no/partly	Standard format was used for documentation n.a./yes/no^1^
Request							
	Unique device identifier	Yes	Yes		Data scientist	Yes	n.a.
	Request form	Yes	Yes		Data scientist	Yes	No
	Requirements	Yes	Yes		Medical doctors	Partly	Yes
Consideration							
	Market exploration	Yes	Yes		Whole team	No	n.a.
Define design criteria		Yes	Yes		Data scientist and medical physicist	No	Yes
Risk analysis							
	Risk analysis ISO14971	Yes	Yes		Whole team	No	Yes
	Risk analysis use	Yes	Yes		Whole team	No	Yes
	Risk management plan	Yes	Yes		Whole team	No	Yes
Design							
	Work Drawing	No	No	Only applicable for physical medical devices.		n.a.	n.a.
	Design process	Yes	Yes			Yes	n.a.
Design verification		Yes	Yes		Data scientist and medical physicist	No	Yes
Prototype							
	Photo	No	No	Only applicable for physical medical devices.		n.a.	n.a.
Define testprotocol		Yes	Yes		Data scientist and medical physicist	Yes	No
Define preconditions							
	List of all preconditions	Yes	Yes		Data scientist and medical physicist	No	Yes
	Training requirements engineers	Yes	No	Training requirements for the engineers (in this case the data scientists) is normally applicable for all medical software, but in this case the standard training requirements of Epic, our EHS, are sufficient, so extra documentation in the technical file was not needed.		n.a.	n.a.
	User training requirements	Yes	Yes		Data scientist, medical physicist and technical physician	No	Yes
	Training engineers	Yes	No	Training for the engineers (in this case the data scientists) is normally relevant for all medical software, but in this case the standard training of Epic, our EHS, was sufficient, so extra documentation in the technical file was not needed.		n.a.	n.a.
	User training	Yes	Yes		Data scientist, medical physicist and technical physician	No	Yes
	Cleaning and sterilization requirements	No	No	Only applicable for physical medical devices.		n.a.	n.a.
	Maintenance prescription	Yes	Yes			Yes	No
	Installation prescription	Yes	Yes		Data scientist	Partly	Yes
	User manual	Yes	Yes		Data scientist, medical physicist and technical physician	Partly	Yes
	Packaging	No	No	Only applicable for physical medical devices.		n.a.	n.a.
	Label	No	No	Only applicable for physical medical devices.		n.a.	n.a.
	Transportation prescription	No	No	Only applicable for physical medical devices.		n.a.	n.a.
Validation		Yes	Yes		Data scientist and medical physicist	Yes	No
Clinical research							
	Research plan	Yes	Yes		Whole team	No	Yes
	Research results	Yes	Yes		Whole team	No	n.a.
Production		Yes	No	Only one item of the prediction model was made, there is no production process.		n.a.	n.a.
Post market surveillance							
	Post market plan	Yes	Yes		Data scientist and medical physicist	Partly	Yes
	Post market reports	Yes	No^2^	When it are only minor deviations, this is documented in the code. When it are major deviations, a new project is started, including all relevant documentation.	Data scientist and medical physicist	n.a.	n.a.
Technical file							
	Technical file	Yes	Yes		Medical physicist and technical physician	Partly	Yes
	Essential requirements	Yes	Yes		Medical physicist and technical physician	No	Yes
	Additional	Yes	No			n.a.	n.a.
Declaration		Yes	Yes		Medical physicist	No	Yes
Takeover protocol		Yes	No	A takeover protocol is normally relevant for all medical software, but in this case the standard protocols of Epic, our EHS, were sufficient, so extra documentation in the technical file was not needed.		n.a.	

^1^ See table 1 for an overview of available formats.

^2^ When it are only minor deviations, this is documented in the code. When it are major deviations, a new project is started, including all relevant documentation.

For the resulting applicable subelements, it was determined that all members of the multidisciplinary team had to contribute to the required documentation, although a large part (7 out of the 22 applicable elements) had to be filled only bythe involved medical physicists and the data scientist (Table 2, column 6).

The standard documentation of data scientists in our hospital consisted of several documents based on the CRISP-DM methodology. Although the standard documentation of the data scientist contained lots of required information, that information was spread over various documents. We decided against transforming it to our QMS formats, but checked if it was complete and fulfilled the requirements of the MDR. For 32% of the applicable documentation, the documentation of the data scientist was sufficient and additional information was not needed (Table 2, column 7), e.g., the validation report, which we added in the supplementary material (Supplementary 3). For 23% the documentation of the data scientist was incomplete and we decided to add relevant information to fulfil the requirements of the MDR and for 45% the documentation of the data scientist was completely lacking and the standard formats had to be used to fulfil the requirements of the MDR.

Because the prediction model was implemented in Epic, our hospital electronic medical record system, some choices were defined beforehand, such as the maintenance schedule, the visual representation of the outcome of the model and basic validation steps after software updates. Extra documentation regarding these items was therefore deemed unnecessary.

The external audit of our completed technical file concluded that the technical file formed a good basisand that the building blocks used from Epic created a safe environment for the development of prediction models. Questions were raised regarding the validation of the software itself and the validation of the results of the software, because relevant standards, such as the IEC62304 and EN82304 were not used. However, the auditor admitted that harmonized standards within the MDR were still very limited.

## Discussion

In general, the QMS intended for the development of physical medical devices was suitable for the development of medical software. Although certain elements are clearly intended for physical devices such as packaging, labelling and transportation requirements, the majority of the elements (83%) is applicable for both software as well as physical devices. This was shown by the high percentage of elements deemed applicable at the start of the project. This observation was valid at the end of the project as well, although a few additional elements were determined not applicable during the project due to the software environment used for development. The difference between the initial estimation of applicability and the final result was small (4 out of 32 items). Two of those items were related to the training of the engineers. It was deemed unnecessary to implement extra training for the engineers because the developers’ environment was within our standard hospital information system environment and training is part of their daily job.

As far as we know, our article is the first one to test if a QMS in place for physical medical devices is also suitable for medical software, whereas other articles focus more on a theoretical framework or challenges regarding the implementation of a QMS (9–11). Most challenges can be reduced by working in a multidisciplinary team, as was done in our situation, and to combine all the knowledge available. Experience with accepting commercial AI tools, development of non-medical software and knowledge of quality systems is mostly available in the larger hospitals, but divided among multiple departments. The model we developed was a so called “type a” AI medical device, which means that it is frozen during its lifetime. This means that only validation before implementation and after changes is necessary, but not continuously during lifetime as is the case with AIMS (8).

The prediction model developed was the first medical software developed by our data science team. The CRISP-DM methodology was used for the development and documentation of this medical software. The different CRISP-DM process steps are also part of the ISO13485, but ISO13485 focuses not only on the development of software, but provides a broader framework for quality management in the organization (Table 2). Therefore, many documents required by the ISO13485 were not in place yet within the data science team. Besides the CRISP-DM methodology, the available documentation is also restricted by the developers’ environment we used. Although development and validation of software is done outside the Epic environment, implementation in the front-end-environmentis bound by Epic guidelines for implementation such as specific model formats, date types and data flow. These guidelines are implemented in the data scientist working method, since they are mandatory to follow to work within the Epic framework. These guidelines can be found on a web portal available to customers of Epic. The documentation the data scientists team delivered is different from the formats we use in the QMS, but was deemed suitable for the QMSsince it contained the same information. The decision of not transforming the CRISP-DM/Epic documentation into the standard formats described in our QMS, made collecting all needed documentation much easier. We therefore concluded that it is important to take in account the intended purpose of an element and not the form in which it is presented. The MDR mandates an appropriate QMS (1) but does not further define this, which gives freedom to adapt it to your own situation. On the contrary, the use of formats does secure compliance of the documents the regulations. In addition deviating from formats may take more time due to the need for detailed analysis of a document withwith respect to the compliance.

When developing in-house medical software, it is dependent on the developers’ environment what information and guidelines are available. Therefore, it is crucial to check the guidelines of the environment before starting software development. If such requirements are not available, the hospital has to define its own guidelines, to make sure that the whole development team is working in the same way and the development fulfills the legal requirements. Standards such as the ISO13485, the IEC62304, and development approaches such as CRISP-DM offer a guideline for such working methods.

The external auditor of our completed technical file raised some question about the validation of the software itself and the validation of the results of the software, because the IEC62304 was not used. The IEC62304 is a norm specifically for the development of medical software, while ISO13485 is a norm for the quality system regarding the development of all types of medical devices. However, all elements of the IEC62304 are incorporated in the ISO13485 (15), including validationv of the medical device and validation of the results andare therefore also part of our QMSQMS (see Table 1). Next to this, validation is also part of the evaluation step in the CRISP-DM methodology. As a hospital, we preferred to have one QMS for all in-house developed medical devices and not different QMSs for different types of medical devices. We already had a QMS in place based on the ISO13485:2016. We therefore decided to investigate if the QMS based on the ISO13485:2016, in combination with the CRISP-DM method, would fulfill all requirements, although the IEC62304 gives more guidance on certain steps in the design and development phase of software than the ISO13485 does. Because the CRISP-DM method gives comparable guidance on these steps (14), IEC62304 explicitly.

In conclusion, we showed in this article that it is possible to use a QMS developed with physical medical products in mind for medical software and thus comply with applicable legislation and regulations. This can be done without too much effort when there is already some structured form of software development methodology in place Development of medical software using the CRISP-DM methodology alone does not fulfill the requirements regarding regulations of medical software. Departments that are developing using this methodology should consider implementing a QMS based on the relevant IEC and/or ISO standards.

## Data Availability

The original contributions presented in the study are included in the article/Supplementary Material, further inquiries can be directed to the corresponding author.

## Supplementary material

Supplementary 1 Format for Design Verification.

Supplementary 2 Format for Post Market Surveillance.

Supplementary 3 Validation report.
