# Supplementary material for: Applying and validating a quality management system for in-house developed medical software
Source: Front Digit Health. 2025 Apr 1;7:1461107. doi: 10.3389/fdgth.2025.1461107 (PMC11996894; doi:10.3389/fdgth.2025.1461107)
Supplement: Supplementary 1 — Format for Design Verification. [file Datasheet1.docx]

**Validation of XGBoost Model: Predicting Fluid Response in ICU**

There are 2 levels at which validation can take place:

1. Global Level
2. Local Level

At the global level, one can mainly look at the importance of variables across the entire dataset. Simply put, the average value for all patients.

At the local level, you look at each patient and how a prediction is made, and what the weight of the variables is in that prediction.

A quick reminder of the performance of the final model:

- Accuracy of the XGB model is 83.22%.
- AUC of the XGB model is 82.99%.
- Precision of the XGB model is 86.21%.
- Recall of the XGB model is 77.38%.
- F1 of the XGB model is 81.56%.

**Global level**

Below is a plot of the 15 most important variables across the entire dataset.
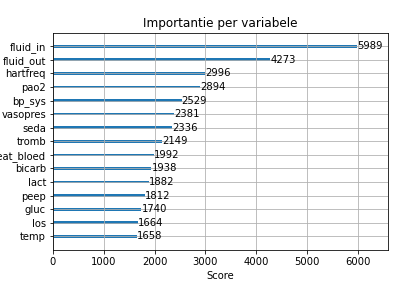


**Local level**

To make a prediction at the local level, we need test patients. Based on Emmy's overview, I randomly selected 5 patients and made a simulation using SHAP (a package in Python).

Selected patients and values:

| MV patientID | 260897 | 260996 | 261034 | 261080 | 261123 |
| --- | --- | --- | --- | --- | --- |
| fluid_in | 1088 | 747 | 1325 | 1079 | 481 |
| fluid_out | 750 | 220 | 985 | 845 | 200 |
| hartfreq | 106 | 86 | 74 | 124 | 70 |
| pao2 | 313 | 41 | 75 | 72 | 87 |
| bp_sys | 110 | 97 | 100 | 147 | 125 |
| vasopres | 0,01 | 0,05 | 0,01 | 0,01 | 0,27 |
| seda | 10 | 22 | 8 | 3002 | 24 |
| creat_bloed | 64 | 69 | 256 | 199 | 77 |
| tromb | 387 | 459 | 186 | 197 | 209 |
| bp_gem | 74 | 67 | 74 | 81 | 89 |
| lact | 4,4 | 3,6 | 1,2 | 1,4 | 1,6 |
| bicarb | 28,8 | 17,8 | 22,0 | 25,2 | 18,2 |
| bp_dia | 60 | 53 | 60 | 53 | 71 |
| temp | 35,0 | 34,7 | 37,2 | 36,7 | 35,0 |
| gluc | 3,9 | 7,8 | 7,1 | 18,1 | 9,3 |
| peep | 8 | 12 | 11 | 12 | 12 |
| Hb | 7,6 | 9,3 | 5,0 | 5,2 | 7,4 |
| los | 57 | 331 | 728 | 1875 | 145 |
| etco2 | 30 | 32 | 34 | 32 | 30 |
| crp | 0,6 | 20,0 | 49,0 | 268,0 | 0,9 |
| albu | 40 | 30 | 31 | 23 | 28 |
| natrium | 40 | 30 | 31 | 23 | 28 |
| Ht | 0,38 | 0,46 | 0,27 | 0,26 | 0,39 |
| opnametype | ongepland | ongepland | gepland | ongepland | ongepland |
| geslacht | vrouw | vrouw | man | man | man |

Predictions for the above patients:

Pat1


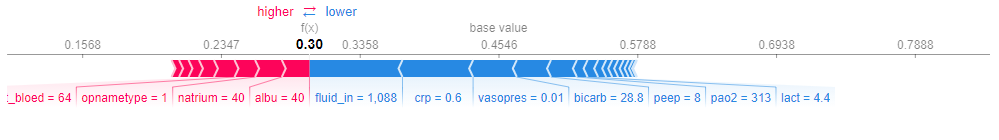


Pat2


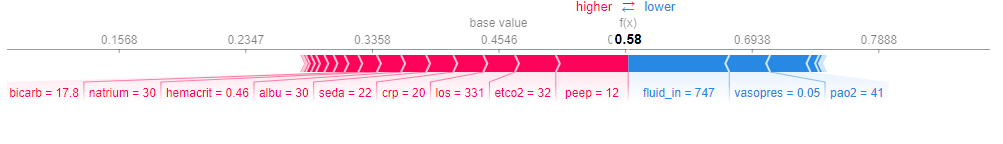


Pat3


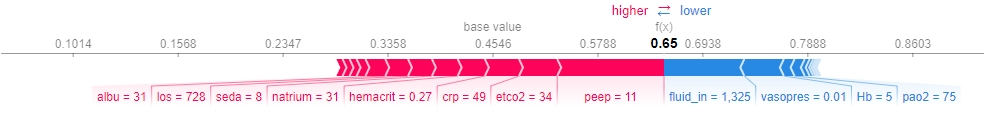


Pat4


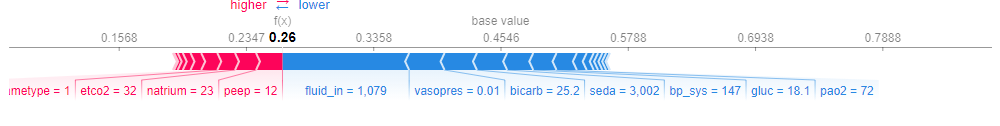


Pat5


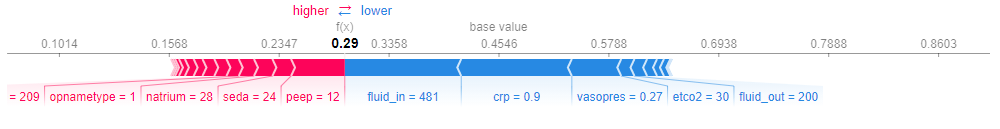


Conclusion: The contribution per variable differs per person and thus contributes to a different predicted probability.

Ultimately, the top number (predicted probability) will be displayed in EPIC, and it is good to determine which probability zones will be used and what actions are associated with them. Two months after go-live is a good time window to choose the precision of the probability zones.

Voorstel zones

0 – 30 Low probability of fluid response

30 – 70 Medium probability of fluid response

70 + High probability of fluid response
