# Supplementary material for: Applying and validating a quality management system for in-house developed medical software
Source: Front Digit Health. 2025 Apr 1;7:1461107. doi: 10.3389/fdgth.2025.1461107 (PMC11996894; doi:10.3389/fdgth.2025.1461107)
Supplement: Supplementary 2 — Format for Post Market Surveillance. [file Datasheet2.docx]

# Format design verification

During the design verification, it must be checked whether the design of the medical device meets the design requirements. Use this format to indicate whether this has been met.

| Medical device: | AI fluid responsiveness model |
| --- | --- |
| Names of those involved in the verification: |  |
| Date of verification: |  |

Tick what applies. The document containing these requirements is indicated in parentheses.

|  | Yes | No | NA | Remarks |
| --- | --- | --- | --- | --- |
| Design meets user needs and requirements. Describe how this was checked. |  |  |  |  |
| Design meets technical specifications. Describe how this was checked. |  |  |  |  |
| Design meets requirements from risk analysis (Risk Analysis). Describe how this was checked. |  |  |  |  |
| Material and/or software choice (operating system, development program, etc.) of the design meets requirements (Risk Analysis). Describe how this was checked. |  |  |  |  |
| Design meets cleaning/sterility requirements (Requirements from DSMH/CSA). Describe how this was checked. |  |  |  |  |
| Design meets other specified design requirements (Design Requirements). Describe how this was checked. |  |  |  |  |
|  |  |  |  |  |
| Verification approved |  |  |  |  |
