# Supplementary material for: Applying and validating a quality management system for in-house developed medical software
Source: Front Digit Health. 2025 Apr 1;7:1461107. doi: 10.3389/fdgth.2025.1461107 (PMC11996894; doi:10.3389/fdgth.2025.1461107)
Supplement: Supplementary 3 — Validation report. [file Datasheet3.docx]

**Post Market Surveillance Plan AI Prediction Model Fluid Responsiveness**

Author: Vera Lagerburg

Version: 1

Date: 7-1-2022

The PMS is conducted semi-annually according to the guidelines set by the EPD service. It has been agreed that for minor changes to the code, the reason and the change in the code will be documented. For major changes, a new project will be started, and the entire process will be repeated.

| **Frequency** | **Date of PMS execution** | **PMS conclusion** | **Adjustment of frequency?** |
| --- | --- | --- | --- |
| Semi-annually |  |  |  |
|  |  |  |  |
|  |  |  |  |
|  |  |  |  |
|  |  |  |  |
|  |  |  |  |
|  |  |  |  |
|  |  |  |  |
